# Dual-guidewire technique with cerebral embolic protection for brachiocephalic trunk bifurcation stenosis: a case report

**DOI:** 10.3389/fcvm.2026.1955380

**Published:** 2026-10-08

**Authors:** Yulan Gan, Guoyin Pang, Guofei Zhang, Jun Yang, Wenyou Wu, Zhifu Song

**Affiliations:** 1 Department of Neonatology, Luzhou People's Hospital, Luzhou, China; 2 Department of Rehabilitation, The Affiliated Hospital of Southwest Medical University, Luzhou, China; 3 Department of Neurosurgery, Luzhou Naxi District People's Hospital, Luzhou, China; 4 Department of Cerebrovascular Disease, Luzhou People's Hospital, Luzhou, China

**Keywords:** brachiocephalic trunk, case report, cerebral embolic protection, dual-guidewire technique, stenting

## Abstract

**Background:**

Severe stenosis at the terminal bifurcation of the brachiocephalic trunk involving the origins of the right common carotid artery and the right subclavian artery is a rare and highly challenging lesion of the supra-aortic vessels. Endovascular intervention carries a high risk of embolization and difficulty in delivering large-diameter stents.

**Case summary:**

We report a 60-year-old male patient who presented with intermittent dizziness and asymmetric blood pressure in the upper limbs. Color Doppler ultrasound and head and neck CT angiography revealed severe stenosis at the terminal bifurcation of the brachiocephalic trunk (stenosis rate approximately 90%), with the lesion involving the origins of the right common carotid artery and the right subclavian artery, accompanied by right subclavian artery steal phenomenon. The patient underwent brachiocephalic trunk balloon angioplasty and stenting via a single femoral artery access. An embolic protection device was deployed in the C1 segment of the right internal carotid artery for distal cerebral protection. During delivery of the stent, the filter guidewire provided insufficient support; a second guidewire was added as a buddy wire to establish a dual-guidewire rail technique, facilitating successful stent placement. Debris was observed in the retrieved filter. Postoperatively, the severe stenosis was relieved and the steal phenomenon resolved. At 1.5 years of follow-up, the patient reported no recurrence of dizziness, and the inter-arm blood pressure difference remained within the normal range.

**Conclusion:**

In this selected case with clear antegrade flow direction, a single-access, single-filter strategy appeared feasible and may offer a simplified alternative for similar anatomical presentations. However, further validation in larger case series is warranted. The application of dual-guidewire rail technique to overcome difficulties in large-diameter stent delivery during brachiocephalic trunk intervention has been rarely reported.

## Introduction

The brachiocephalic trunk (BCT) terminates by dividing into the right common carotid artery and the right subclavian artery. Isolated severe stenosis at this bifurcation involving the origins of both branches is rarely encountered in clinical practice. Interventional treatment of such lesions faces dual challenges: debris dislodged during balloon angioplasty and stent deployment can simultaneously threaten the anterior circulation (via the internal carotid artery) and the posterior circulation (via the vertebral artery), resulting in a high risk of intraoperative stroke; additionally, the large vessel caliber at this site demands substantial guidewire support for delivery of large-diameter stent systems.

According to current vascular guidelines, endovascular intervention is recommended for symptomatic innominate artery lesions, while asymptomatic lesions do not require intervention (1, 2). Innominate artery stenosis is a rare clinical entity, with an incidence of approximately 0.65% reported in an unselected population of 2,768 patients (3).

In this report, we describe a case of isolated severe stenosis at the terminal bifurcation of the brachiocephalic trunk, managed with a single femoral access, single-filter cerebral embolic protection, and a dual-guidewire rail technique to overcome insufficient guidewire support during large-diameter stent delivery.

## Case report

### Patient information

A 60-year-old male patient was admitted on January, 2025, with complaints of “intermittent dizziness for over 1 month.” Four days prior to admission, a health check-up revealed asymmetric blood pressure between the upper limbs, with a difference of approximately 30 mmHg. Physical examination: T 36 °C, P 75 beats/min, R 20 breaths/min, blood pressure 144/96 mmHg in the right upper limb and 184/81 mmHg in the left upper limb. The right radial pulse was significantly weaker compared to the contralateral side. Past medical history included long-term smoking (approximately 20 cigarettes per day), with occasional alcohol consumption.

### Diagnostic assessment

Upper extremity vascular Doppler ultrasound (January, 2025): The right innominate artery showed multiple plaques, the largest located on the anterior wall measuring approximately 15 mm in length and 7.7 mm in thickness, causing severe luminal stenosis with an original diameter of 7.7 mm and a residual lumen of 1.6 mm, with a peak systolic velocity of 227 cm/s at the stenosis. The right subclavian artery showed multiple plaques, the largest measuring approximately 22 mm × 4.4 mm. The triphasic waveform of the right upper extremity artery was absent. Transcranial Doppler revealed right vertebral artery steal phenomenon.

Head and neck CT angiography: A localized severe stenosis was identified at the terminal bifurcation of the brachiocephalic trunk, involving the origins of the right common carotid artery and the right subclavian artery, with a diameter stenosis rate of approximately 90% (Figures 1A–C).

**Figure 1:**
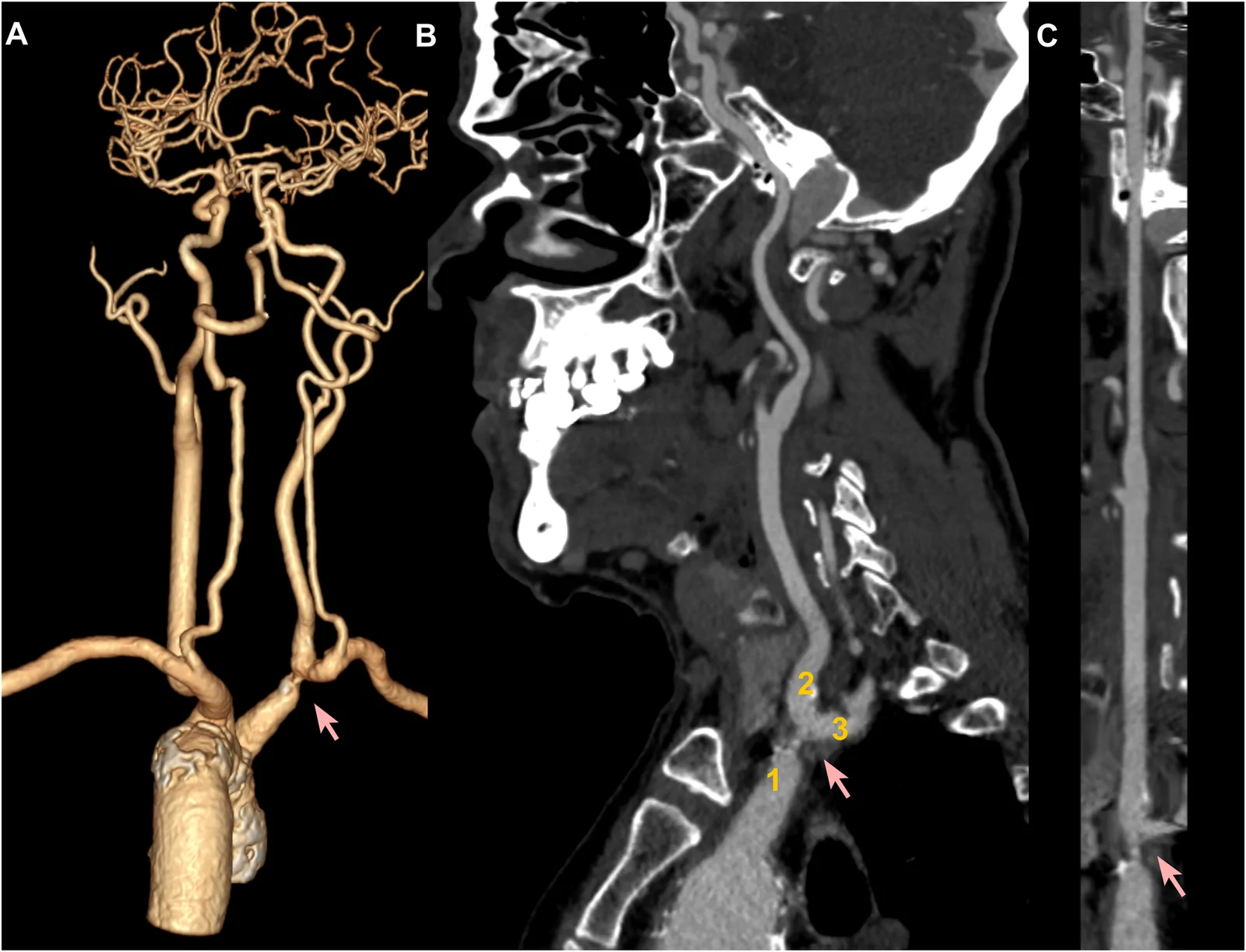
Preoperative head and neck CT angiography. **(A)** Head and neck CTA showing severe stenosis at the terminal bifurcation of the brachiocephalic trunk (pink arrow), with the lesion involving the right subclavian artery and the origin of the right common carotid artery. The plaque was of mixed density (partially calcified), measuring approximately 15 mm in length. **(B)** Raw image showing atherosclerotic plaque causing severe stenosis at the bifurcation (pink arrow), 1 = distal brachiocephalic trunk, 2 = right subclavian artery, 3 = origin of the right common carotid artery. **(C)** MIP image showing bifurcation stenosis (pink arrow). Stent deployment at the origin of the right common carotid artery can effectively improve flow to both the right common carotid artery and the right subclavian artery, obviating the need for dual stents. Delayed opacification of the right vertebral artery was observed, suggestive of subclavian artery steal phenomenon.

Diagnoses: (1) Severe stenosis at the terminal bifurcation of the brachiocephalic trunk (involving the origins of the right common carotid artery and the right subclavian artery); (2) Hypertension grade 3 (very high risk); (3) Right subclavian artery steal syndrome.

### Interventional treatment

After obtaining informed consent, brachiocephalic trunk balloon angioplasty and stenting was performed. Right femoral artery access was obtained with an 8F vascular sheath, and a guide catheter was advanced to the proximal stenosis. Cerebral angiography confirmed severe stenosis at the terminal bifurcation of the brachiocephalic trunk (Figure 2A). Preoperative left vertebral artery angiography demonstrated significant right vertebral artery steal (Figure 2B).

**Figure 2:**
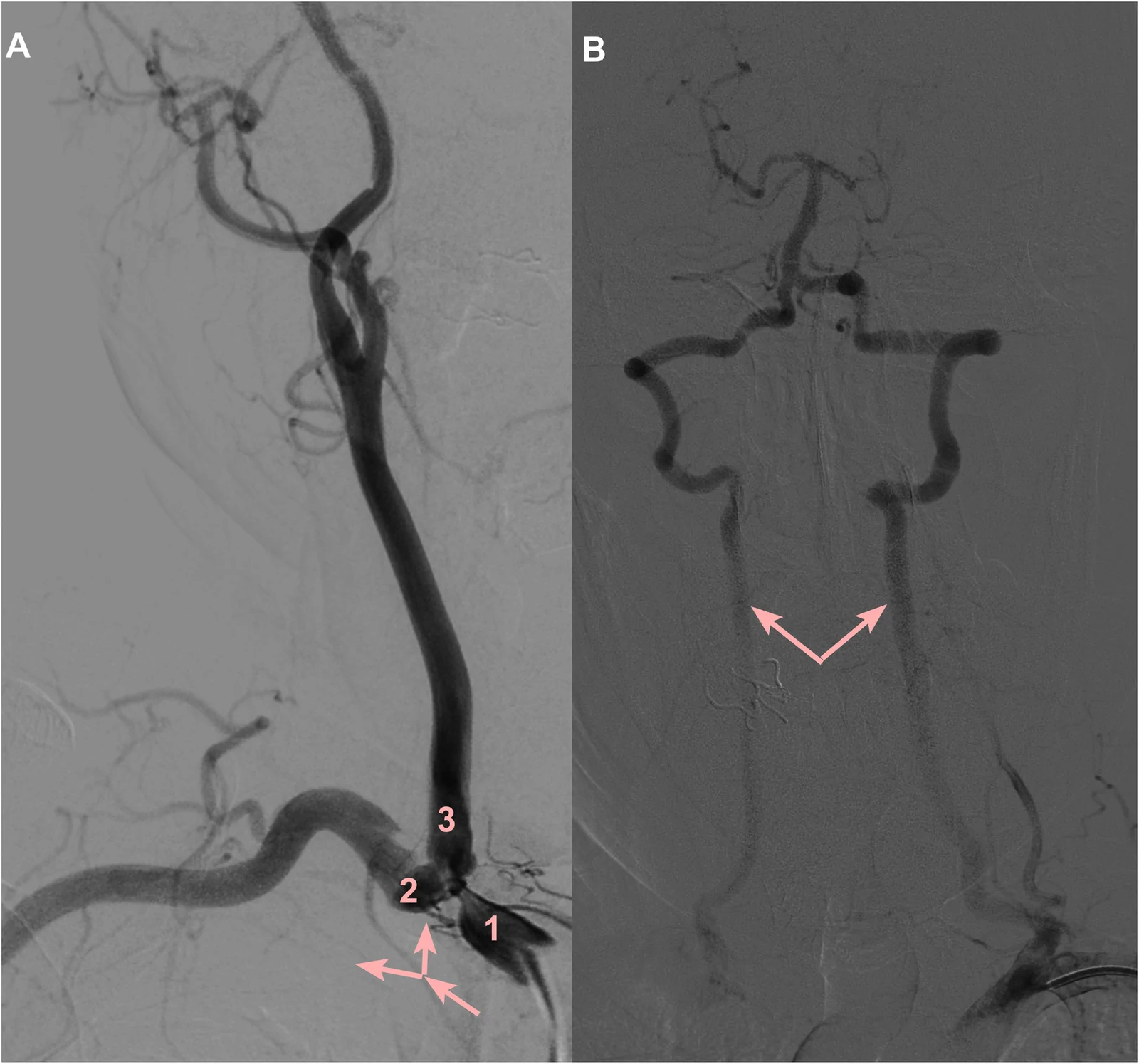
Intraoperative cerebral angiography. **(A)** Intraoperative angiography showing severe stenosis at the terminal bifurcation of the brachiocephalic trunk. The three pink arrows indicate the direction of blood flow from the brachiocephalic trunk (1) to the right common carotid artery (2) and the right subclavian artery (3), showing significantly less contrast opacification in the subclavian artery compared to the carotid artery, with no opacification of the right vertebral artery. **(B)** Left vertebral artery angiography (left and right pink arrows) showing significant right vertebral artery steal.

The decision to place a single filter in the right ICA was based on intraoperative angiography demonstrating that antegrade flow was predominantly directed toward the carotid territory, while the right vertebral artery exhibited retrograde flow (subclavian steal phenomenon). In this hemodynamic setting, the risk of embolic material traveling to the vertebrobasilar territory was considered minimal. Yanagihara et al. reported a similar strategy, successfully performing retrograde brachiocephalic artery stenting using a single filter for ICA protection while relying on the increased subclavian steal phenomenon for hemodynamic protection of the vertebral artery (4).

In subclavian steal syndrome, the reversed flow in the vertebral artery creates a natural protective mechanism against antegrade embolization. Ringelstein et al. demonstrated that even after angioplasty, the flow reversal in the vertebral artery does not immediately convert to antegrade flow but gradually reverses within 20 s to several minutes, which serves as a protective mechanism against cerebral embolism (5). Therefore, in the presence of documented vertebral artery flow reversal, the incremental benefit of additional vertebral protection was considered limited.

Given the heavy plaque burden and high embolic risk, an embolic protection device was deployed in the straight segment of the right internal carotid artery (C1 segment) via a microguidewire (Figure 3A). After predilation with a 4 mm × 30 mm balloon, the Express LD stent (8 mm × 27 mm) was advanced over the 0.014-inch filter guidewire. During advancement, the 0.014-inch filter guidewire provided insufficient support for the large-diameter stent system. A second 0.018 ControlWire guidewire was therefore added as a buddy wire (Figure 3A), establishing a dual-guidewire rail technique to assist stent positioning (the filter was noted to have mild migration intraoperatively, but this did not compromise protection). Residual stenosis persisted after stent deployment (Figure 3B), A non-compliant balloon (4 mm × 30 mm) was advanced over the dual-guidewire system to the residual stenotic segment within the stent and inflated to 14 atm, maintained for approximately 10 s. This post-dilation fully resolved the residual stenosis (Figure 3C). The delivery system, guidewires, and filter were sequentially withdrawn, and debris was observed in the retrieved filter. Final angiography demonstrated complete resolution of the severe stenosis, with antegrade flow restored in the right subclavian artery and right common carotid artery, opacification of the vertebral artery (Figure 3C), and resolution of the right vertebral artery steal on left vertebral artery angiography (Figure 3D). The patient remained neurologically intact postoperatively with symmetric bilateral radial pulses.

**Figure 3:**
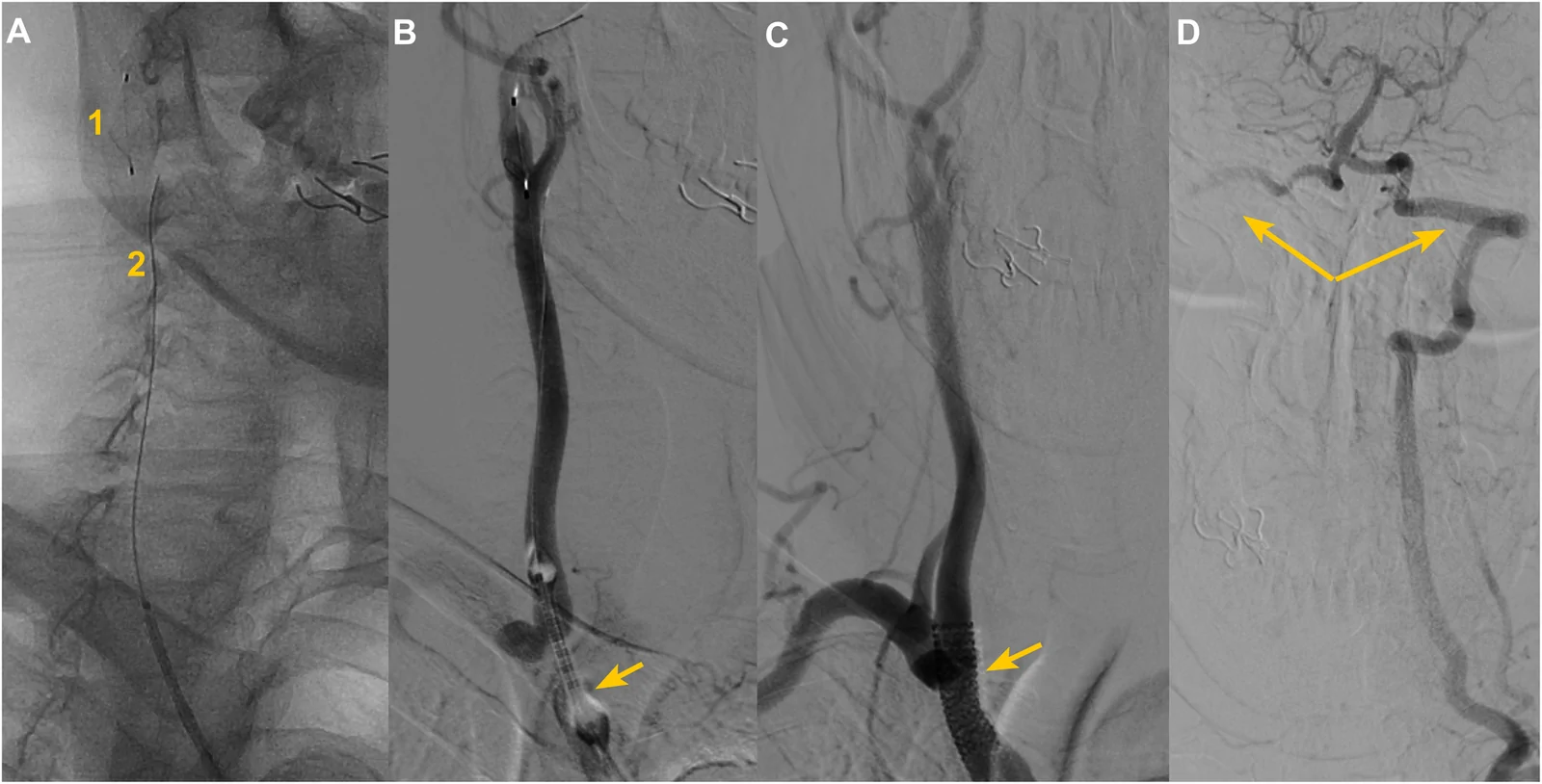
Intraoperative procedure and post-procedural results. **(A)** Intraoperative dual-guidewire rail technique: 1 = distal filter and its associated guidewire (0.014 inch), 2 = V-18 guidewire (0.018 inch). The dual-guidewire technique with distal filter was used to advance the stent to the stenosis (mild filter migration was observed intraoperatively). **(B)** Balloon dilation of the stent, showing residual stenosis. **(C)** After stent deployment, stenosis resolved and the right vertebral artery became opacified. **(D)** Left vertebral artery angiography showing resolution of right vertebral artery steal.

Procedural details: Intravenous heparin was administered (3000 U bolus; as the procedure duration did not exceed 1 h, no additional heparin was given). Activated clotting time was not monitored. No vasodilators were used during the procedure. Fluoroscopy time was 40 min, contrast volume was 120 mL, and radiation dose (air kerma) was 1,245 mGy.

Neurological assessment: Independent neurological assessment was performed by a neurologist both immediately after the procedure and at 24 h post-procedure, with no new deficits detected.

Filter debris analysis: The debris captured in the filter was visually inspected and confirmed to be particulate embolic material; no formal histopathological analysis was performed, as this was not part of the routine clinical protocol.

### Follow-up and outcomes

Duplex ultrasound performed on postoperative day 1 revealed a clear stent echo within the right innominate artery with patent stent flow; the right subclavian artery plaque had reduced to 9.2 mm × 2.0 mm; the right axillary, brachial, radial, and ulnar arteries demonstrated normal diameter and course with normal flow signals and restored triphasic waveforms. The patient's dizziness resolved completely, and upper limb blood pressures were symmetric (right upper limb 138/82 mmHg, left upper limb 140/84 mmHg). The patient was discharged on postoperative day 3.

Clopidogrel was discontinued at 3 months postoperatively, while aspirin (100 mg once daily), atorvastatin (40 mg once daily at bedtime), and ezetimibe (10 mg once daily at bedtime) were continued. At 1.5 years of follow-up, the patient reported no recurrence of dizziness, with inter-arm blood pressure difference remaining within the normal range, and quality of life and functional capacity fully restored. While routine CTA or duplex ultrasound at 12–18 months was not performed (as the patient remained asymptomatic with symmetric blood pressures, and the patient declined further imaging due to significant symptom relief confirmed by telephone follow-up), continued clinical surveillance is ongoing.

The complete timeline of the patient's clinical course is summarized in Table 1.

**Table 1:** Timeline of care.

Time Point	Event	Clinical/Imaging Findings	Management
January 2025	Admission	Right upper limb BP 144/96 mmHg, left upper limb BP 184/81 mmHg	—
January 2025	Doppler ultrasound	Innominate artery original diameter 7.7 mm, residual lumen 1.6 mm, PSV 227 cm/s	—
January 2025	CTA	Terminal BCT bifurcation stenosis ∼90%, involving right CCA + right subclavian origins	—
January 2025	Intervention	—	Filter in right ICA; dual-guidewire rail technique; Express LD stent; post-dilation resolved stenosis
Postop day 1	Doppler ultrasound	Stent patent, right subclavian artery plaque reduced to 9.2 mm × 2.0 mm, triphasic waveform restored	—
Postop day 3	Discharge	Dizziness resolved, symmetric upper limb BP, no neurological deficits	—
Postop 3 months	Medication	—	Clopidogrel discontinued; aspirin + atorvastatin + ezetimibe continued
Postop 1.5 years	Follow-up	No dizziness recurrence, inter-arm BP difference normal	—

## Discussion

This case demonstrates successful endovascular treatment of severe stenosis at the terminal bifurcation of the brachiocephalic trunk, with three key technical features that distinguish it from previously reported cases.

Current evidence regarding cerebral embolic protection in supra-aortic interventions remains limited, and no universally established standard exists. The ESVS 2023 guidelines do not provide a specific recommendation on the use of embolic protection devices during brachiocephalic trunk interventions (1), reflecting the ongoing uncertainty in this area.

Several case reports and small series have described endovascular treatment of brachiocephalic trunk stenosis with various cerebral protection strategies, including single-filter protection, dual-filter protection via brachial or radial artery access, and dual neuroprotective filters (6–9). However, most of these approaches require multi-access or multi-device setups, increasing procedural complexity.

The novelty of our approach lies not merely in the rarity of the case, but in the technical decision-making: the dual-guidewire rail technique represents a practical solution to a specific technical challenge (insufficient guidewire support during large-diameter stent delivery) that has been rarely described in brachiocephalic trunk interventions. This approach is presented as an additional strategy in the context of existing guideline-directed interventions, not as a replacement.

### Highlight 1: precise anatomical localization

Most previously reported cases of brachiocephalic trunk stenosis have focused on lesions at the origin or mid-portion of the vessel. Onishi et al. (8) reported stenting for innominate artery stenosis but did not specify that the lesion was located at the terminal bifurcation. Sadiq et al. (9) reported brachiocephalic artery stenting with dual-filter protection, but the lesion was similarly located in the mid-portion. Cases specifically described as “isolated severe stenosis at the terminal bifurcation of the brachiocephalic trunk involving the origins of both the right common carotid artery and the right subclavian artery” are rarely reported in the literature. This precise anatomical localization represents the primary unique value of our case. The CTA and intraoperative angiography clearly demonstrate this anatomical feature (Figures 1A–C, 2A), providing a reference for imaging recognition of similar cases in the future.

### Highlight 2: single-access, single-filter strategy

Cerebral protection strategies for brachiocephalic trunk intervention reported in the literature have predominantly employed complex multi-access approaches: Onishi et al. (8) performed stenting via the right femoral artery with two filters placed via the right brachial artery (one in the right internal carotid artery and one in the right vertebral artery); Sakamoto et al. (10) performed simultaneous distal balloon protection of the right internal carotid artery and vertebral artery via the right brachial artery; Irvine et al. (7) used dual neuroprotective filters. The common feature of these strategies is the requirement for more than one access site or more than one protection device, resulting in complex procedures and increased invasiveness.

In contrast, our case was successfully completed through a single right femoral artery access with a single filter placed in the right internal carotid artery. The success of this simplified strategy was based on the intraoperative determination that antegrade flow was predominantly directed toward the carotid territory (Figure 2A shows flow predominantly toward the internal carotid artery), while the right vertebral artery flow represented reverse flow from the left vertebral artery. Tsygankov et al. (6) reported 5 cases of brachiocephalic trunk intervention using the Spider FX filtering protective device, achieving 100% angiographic and clinical success with debris captured in all cases, supporting the feasibility of the single-filter strategy. Debris was also observed in the retrieved filter in our case. Our case suggests that when the direction of blood flow is clear—specifically, when antegrade flow is predominantly toward the internal carotid artery and the right vertebral artery exhibits reverse flow—a single-access, single-filter strategy may provide sufficient cerebral protection while avoiding the additional trauma and procedural time associated with multi-access approaches.

### Highlight 3: dual-guidewire rail technique

A notable technical aspect of this case is the application of the dual-guidewire rail technique in brachiocephalic trunk intervention. When the 0.014-inch filter guidewire provided insufficient support for delivery of the 8 mm large-diameter stent, a second 0.018-inch ControlWire guidewire was placed into the external carotid artery as a buddy wire to establish a dual-guidewire rail (Figure 3A), increasing support and enabling successful stent delivery. The V-18 guidewire features an ICE hydrophilic coating and a Scitanium stainless steel core, designed for supporting balloon catheters and other therapeutic devices in peripheral vascular interventions; in this case, it served as a buddy wire providing additional rail support.

The dual-guidewire/buddy wire technique has been well established in complex coronary interventions, but reports of its application in brachiocephalic trunk interventions are limited. Our case extends this strategy to large-diameter stent delivery for stenosis at the terminal bifurcation of the brachiocephalic trunk, demonstrating the applicability of the dual-guidewire rail technique in supra-aortic large-vessel interventions. Although the filter exhibited mild migration during the procedure (Figure 3A), protection was not compromised, highlighting the importance of closely monitoring filter position during manipulation.

Potential risks of the buddy-wire technique deserve consideration, including: vessel injury or dissection from the second guidewire, guidewire entanglement, filter displacement or migration (which we observed as mild migration in our case), embolization during guidewire exchange, and risk of stent malposition. In our case, these risks were mitigated by careful guidewire manipulation, continuous fluoroscopic monitoring, and maintaining the buddy wire within the external carotid artery to avoid interference with the filter.

### Comparison with previously reported cases

Comparing our case with previously reported brachiocephalic trunk intervention cases: Irvine et al. (7) used dual neuroprotective filters (dual access); Sakamoto et al. (10) used dual balloon occlusion protection (via the brachial artery); Onishi et al. (8) used dual-filter protection (femoral + brachial artery dual access); Sadiq et al. (9) used a dual-filter embolic protection device (via the radial artery). All of the above reports employed multi-access or multi-device strategies, whereas our case was completed through a single-access, single-filter approach. Furthermore, none of the above reports mentioned the use of dual-guidewire technique to address stent delivery challenges, and descriptions of dual-guidewire rail technique application in brachiocephalic trunk intervention are limited.

The key features of previously reported cases and our case are summarized in Table 2.

**Table 2:** Comparison of the present case with previously reported cases.

Study	Lesion Location	Access	Protection	Key Outcomes
Tsygankov et al. (2017) (6)	Brachiocephalic trunk (unspecified)	Not specified	Spider FX (single filter)	5 patients, 100% success, debris captured
Irvine et al. (2025) (7)	Brachiocephalic artery (unspecified)	Not specified	Dual neuroprotective filters	Elderly female, symptom-free at 6 weeks
Onishi et al. (2020) (8)	Innominate artery (unspecified)	Femoral + Brachial	Dual filters (ICA + VA)	58F, symptom resolved, no complications
Sadiq et al. (2020) (9)	Brachiocephalic artery (unspecified)	Radial	Sentinel dual-filter	77F, right arm claudication
Sakamoto et al. (2013) (10)	Innominate artery (unspecified)	Femoral + Brachial	Distal balloon (ICA + VA)	82M, IA stenosis with infarction, success
Current case	BCT terminal bifurcation (CCA + Subclavian origins)	Single femoral	Single filter (ICA only)	60M, single-access single-filter, dual-guidewire rail, 1.5-year symptom-free

ICA, internal carotid artery; VA, vertebral artery; CCA, common carotid artery; IA, innominate artery; BCT, brachiocephalic trunk; F, female; M, male.

In addition, residual stenosis was observed after initial stent deployment in our case, which was completely resolved after adequate post-dilation (Figures 3B,C comparison), suggesting that for bifurcation lesions with heavy plaque burden, the angiographic result after initial stent deployment should not be considered sufficient, and adequate post-dilation is critical to ensure optimal stent apposition.

### Limitations

This study is a case report with the following limitations: (1) Single-case observation limits the generalizability of conclusions; (2) Whether the single-filter strategy is applicable to all patients with lesions involving both branches requires individualized decision-making based on intraoperative blood flow direction and is not suitable for all brachiocephalic trunk bifurcation lesions; (3) The generalizability of the dual-guidewire rail technique requires validation in more cases. Accumulation of additional cases is needed to further validate the general applicability of this strategy.

### Clinical takeaways

This case offers the following practical clinical observations: (1) For stenosis at the terminal bifurcation of the brachiocephalic trunk, a single-access, single-filter strategy appeared feasible in a carefully selected patient with clear antegrade flow direction; (2) When large-diameter stent delivery is hampered by insufficient guidewire support, the dual-guidewire rail technique may be a useful solution; (3) For bifurcation lesions with heavy plaque burden, adequate post-dilation is important to ensure optimal stent apposition.

## Patient perspective

The patient's dizziness resolved completely after the procedure, and upper limb blood pressures returned to symmetry (right upper limb 138/82 mmHg, left upper limb 140/84 mmHg on postoperative day 1), with significant improvement in functional capacity. At 1.5 years of follow-up, the patient reported no recurrence of symptoms, with markedly improved quality of life, and expressed great satisfaction with the treatment outcome.

## Data Availability

The original contributions presented in the study are included in the article/Supplementary Material, further inquiries can be directed to the corresponding authors.
